# Efficacy of carbapenem vs non carbapenem β-lactam therapy as empiric antimicrobial therapy in patients with extended-spectrum β-lactamase-producing *Enterobacterales* urinary septic shock: a propensity-weighted multicenter cohort study

**DOI:** 10.1186/s13613-023-01106-z

**Published:** 2023-03-24

**Authors:** Erwann Cariou, Romain Griffier, Arthur Orieux, Stein Silva, Stanislas Faguer, Thierry Seguin, Saad Nseir, Emmanuel Canet, Arnaud Desclaux, Bertrand Souweine, Kada Klouche, Olivier Guisset, Jerome Pillot, Walter Picard, Tahar Saghi, Pierre Delobel, Didier Gruson, Renaud Prevel, Alexandre Boyer

**Affiliations:** 1 Medical Intensive Care Unit, CHU de Bordeaux, 33000 Bordeaux, France; 2 Department of Public Health, University of Bordeaux, 33000 Bordeaux, France; 3 Centre de Recherche Cardio-Thoracique de Bordeaux, Inserm UMR 1045, University Bordeaux, 33000 Bordeaux, France; 4 Intensive Care Unit, University Hospital of Purpan, 31300 Toulouse, France; 5 Intensive Care Unit, Department of Nephrology and Organ Transplantation, Centre for Rare Renal Diseases, University Hospital of Toulouse, 31000 Toulouse, France; 6 Intensive Care Unit, University Hospital of Rangeuil, 31000 Toulouse, France; 7 Department of Intensive Care Medicine, Critical Care Center, CHU of Lille, 59000 Lille, France; 8 Medical Intensive Care Unit, Nantes University Hospital, 44000 Nantes, France; 9 Infectious and Tropical Diseases Department, CHU Bordeaux, 33000 Bordeaux, France; 10 Medical Intensive Care Unit, Gabriel-Montpied University Hospital, 63000 Clermont-Ferrand, France; 11 Medical Intensive Care Unit, CHU Montpellier, 34000 Montpellier, France; 12 Intensive Care Unit, Hôpital Saint-Léon, Centre Hospitalier de la Côte Basque, 64100 Bayonne, France; 13 Intensive Care Unit, Centre Hospitalier de Pau, 64000 Pau, France; 14 Intensive Care Unit, Polyclinique Bordeaux Nord Aquitaine, 33000 Bordeaux, France; 15 Infectious and Tropical Diseases Department, CHU Toulouse, 31000 Toulouse, France

**Keywords:** Extended-spectrum β-lactamase-producing *Enterobacterales*, Non carbapenem β-lactam therapy, Antimicrobial resistance, Septic shock, Urinary tract infection, Aminoglycosides

## Abstract

**Background:**

The rise in antimicrobial resistance is a global threat responsible for about 33,000 deaths in 2015 with a particular concern for extended-spectrum beta-lactamase-producing *Enterobacterales* (ESBL-E) and has led to a major increase in the use of carbapenems, last-resort antibiotics.

**Methods:**

In this retrospective propensity-weighted multicenter observational study conducted in 11 ICUs, the purpose was to assess the efficacy of non carbapenem regimen (piperacillin–tazobactam (PTZ) + aminoglycosides or 3rd-generation cephalosporin (3GC) + aminoglycosides) as empiric therapy in comparison with carbapenem in extended-spectrum β-lactamase-producing *Enterobacterales* (ESBL-E) urinary septic shock. The primary outcome was Day-30 mortality.

**Results:**

Among 156 patients included in this study, 69 received a carbapenem and 87 received non carbapenem antibiotics as empiric treatment. Baseline clinical characteristics were similar between the two groups. Patients who received carbapenem had similar Day-30 mortality (10/69 (15%) vs 6/87 (7%), OR = 1.99 [0.55; 5.34] *p* = 0.16), illness severity, resolution of septic shock, and ESBL-E infection recurrence rates than patients who received an empiric non carbapenem therapy. The rates of secondary infection with *C. difficile* were comparable.

**Conclusions:**

In ESBL-E urinary septic shock, empiric treatment with a non carbapenem regimen, including systematically aminoglycosides, was not associated with higher mortality, compared to a carbapenem regimen.

**Supplementary Information:**

The online version contains supplementary material available at 10.1186/s13613-023-01106-z.

## Background

The rise in antimicrobial resistance is a major threat worldwide [1]. The spread of ESBL-E has led to a major increase in the use of carbapenems resulting in the emergence of carbapenem-resistant *Enterobacterales* (CRE). It is therefore essential to avoid the use of carbapenems as much as possible [2–5]. ESBL-E mostly remain susceptible to piperacillin-tazobactam (about 85% of ESBL-producing *Escherichia coli* isolates and 70% of ESBL-producing *Klebsiella pneumoniae*) [6] and to certain aminoglycosides, especially amikacin (about 87% of both ESBL-producing *E. coli* and *K. pneumoniae*) [7]. Regimen, including those molecules, should be investigated as alternatives to carbapenems [8–10].

Regarding severe ESBL-E infections, a retrospective, multicenter study conducted in 2019 [11], including about 100 patients in ICU, found no significant difference between a beta-lactam–beta-lactamase inhibitor (βL–βLI) combination and carbapenem regarding Day-30 mortality but only 6% of these infections were urinary tract ones. The randomized international multicenter MERINO trial [12] did not achieve to demonstrate the non-inferiority of PTZ compared to meropenem regarding the mortality at Day-30 in the treatment of 3GC-resistant *E. coli* and *K. pneumoniae* bloodstream infections but UTI was present only in 60% of the patients. Regarding urinary tract infections [13–15], the third leading cause of septic shock, aminoglycosides are of particular interest as carbapenem-sparing candidates due to their excellent diffusion in the urinary tract [16]. A recent study confirmed this potential interest but included only 10% of septic shock [17]. Despite those encouraging results, current recommendations strongly recommend the use of carbapenems as empiric treatment in urinary septic shock patients with risk factors for ESBL-E infections [18, 19].

The aim of this study is thus to compare the efficacy of carbapenem vs non carbapenem therapy as empiric antimicrobial therapy in ESBL-E urinary septic shock patients.

## Methods

### Study design and inclusion criteria

The SCRUTIN study is a retrospective cohort study conducted in 11 French intensive care units (ICU) from 8 teaching hospitals and 3 general hospitals. Every patient admitted to ICU for septic shock of urinary tract origin between January, 2014 and December, 2020 was considered for inclusion. Septic shock was defined according to the SEPSIS III definition. ESBL-E urinary tract infection was diagnosed by isolation of bacteria from urine culture with antimicrobial susceptibility testing performed with Vitek 2 instrument (bioMérieux^®^, Marcy l’Étoile, France). ESBL production was defined as resistance to one or more oxyimino cephalosporins (e.g., ceftazidime, ceftriaxone, cefotaxime) and confirmed by culture thanks to ESBL Etest (bioMérieux^®^) [20]. Bacterial species was further identified by MALDI Biotyper^®^, Bruker, Bremen, Germany. Patients were included if they received a carbapenem or a non carbapenem agent (3GC + amikacin or PTZ + amikacin) as empiric therapy. For patients with multiple episodes of urinary septic shock due to ESBL-E, only the first episode was considered. Non-inclusion criteria were infection with a Carbapenem-resistant *Enterobacterales*, a suspected associated source of infection, and decisions of withholding or withdrawing intensive therapies.

### Data collection

Data were retrospectively collected from the electronic medical records and electronic worksheet was completed by two medical intensive care investigators.

Comorbidities were defined as follows: chronic obstructive pulmonary disease and asthma were defined according to lung function testing. Chronic heart failure was defined according to transthoracic echocardiography and chronic coronary disease based on stress test or percutaneous coronary intervention. Other comorbidities included history of chronic kidney disease (glomerular filtration rate < 60 mL/min/1.73 m^2^), immunosuppression (drugs, hematological disease, blood marrow transplantation, solid organ transplantation, plasma exchanges indicated by autoimmune disorders, human immunodeficiency virus infection), Charlson score, and simplified acute physiology score II (SAPSII). Acute respiratory distress syndrome (ARDS) was defined according to Berlin’s criteria and AKI to KDIGO guidelines.

Nosocomial acquisition was considered when symptoms of infection started over 48 h after hospital admission or within 48 h of hospital discharge, whereas we considered healthcare-associated acquisition if patients had attended hemodialysis or received intravenous chemotherapy in the past 30 days, had been admitted to an acute-care hospital for at least 2 days or had surgery in the past 90 days, or resided in a nursing home or long-term care facility. Other infections were considered community acquired.

In this retrospective study, no recommendation was made to physicians. However, to guide the collection of data, the risk factors for infection with an ESBL-E considered for the choice of carbapenems were, within the 3 previous months, the use of 2GC, 3GC, amoxicillin–clavulanic acid or fluoroquinolones, a trip in an endemic area, hospitalization, history of UTI, or colonization with ESBL-E for community patients, according to current recommendations [21]. In healthcare-associated infections, prolonged hospitalization in a long-stay facility and the presence of an indwelling catheter or gastrostomy are additional factors. The susceptibilities of bacterial isolates were defined according to EUCAST guidelines [20], i.e., susceptibility to PTZ (MIC ≤ 8 mg/L) and to aminoglycosides (MIC ≤ 8 mg/L for amikacin, ≤ 2 mg/L for gentamicin). Recurrence of an ESBL-E infection, occurrence of a CRE infection, or a *Clostridioides difficile* infection could only be investigated during the current hospitalization.

### Outcomes

The primary efficacy outcome was death from any cause at Day 30 after admission to ICU.

Secondary outcomes were in-ICU mortality, Day-90 mortality, ICU and hospital length of stay, illness severity defined by the duration and maximum dose of norepinephrine, use of mechanical ventilation, use of renal replacement therapy, and time to clinical cure which was defined as the first day at which the patient experienced resolution of septic shock (norepinephrine withdrawal) + resolution of sepsis according to clinical (resolution of fever) and biological criteria (decrease by at least twofold of the leukocyte count).

### Statistical analyses

No statistical sample size calculation was performed a priori, and sample size was equal to the number of patients admitted to ICU with urinary septic shock due to ESBL-E during the study period. Qualitative variables were described with frequencies and proportions. Quantitative variables were described with frequencies, mean, standard deviation, median, minimum, maximum, 1st, and 3rd quartiles. The quantitative variables were compared by Student *t* test if the conditions of validity were respected (normal distribution, homogeneous variances) or Student *t* test for unequal variances in other cases; if the distribution was not normal, the non-parametric Wilcoxon test was used. The qualitative variables were compared with a Chi^2^ test, or corrected Chi^2^ test, or with non-parametric Fisher’s exact test, according to the size of the expected values under the hypothesis of independence. All statistical tests were 2-tailed and statistical significance was defined as *p* < 0.05.

Prescribing carbapenem or a non carbapenem solution in urinary tract-onset septic shock could be related to a patient severity profile. Thus, physicians might have a propensity to treat more severe patients with carbapenems and less severe patients with a non carbapenem regimen. We therefore constructed a propensity score for receiving carbapenems in the empiric treatment of urinary septic shock. For this purpose, we performed a univariate and then a multivariate logistic regression of the risk of being treated with carbapenems compared to an alternative. The explanatory variables retained were age, sex (male), Charlson index, cardiovascular history, immunosuppression status, presence of risk factors for ESBL-E infection, SOFA score, and obstructive pyelonephritis. The propensity score of each patient was then integrated into the multivariate model analyzing the risk factors of mortality at Day-30 to adjust for the impact of the empiric treatment. The treatment effect was estimated by a weighted logistic regression model. The parameters of the model were estimated by bootstrapping. The balancing property of propensity score was assessed graphically using standardized differences before and after ponderation by weighting.

### Ethics

The study obtained the approval of the Institutional Review Board of the Bordeaux University Hospital (CER-BDX-2022-02). This database is registered by the Commission Nationale Informatique et Libertés (CNIL, Registration no 2222361v0.).

## Results

**Fig. 1:**
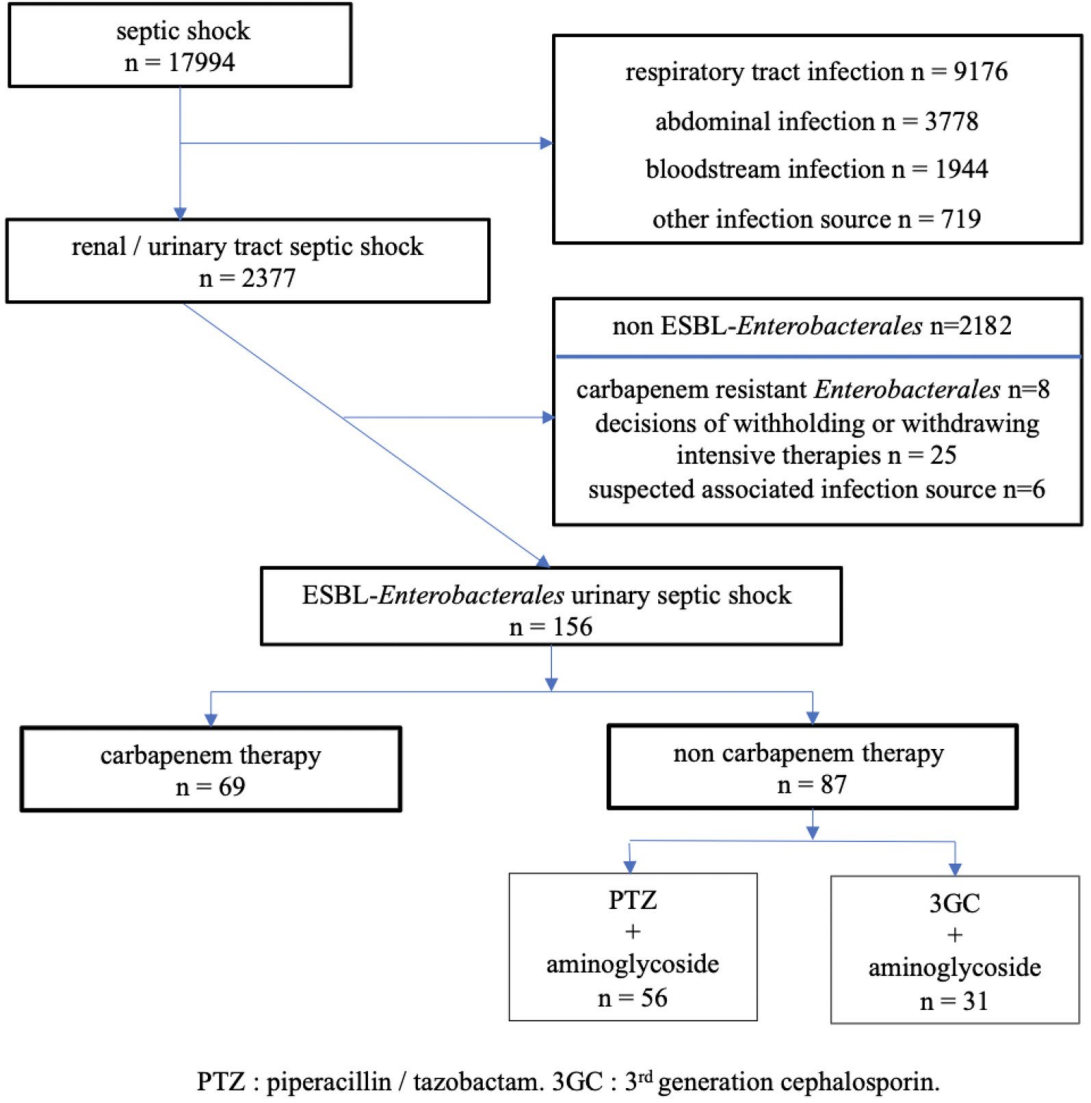
Flow chart

One hundred and fifty-six patients admitted to ICU for ESBL-E urinary septic shock between January 2014 and December 2020 were retrospectively included, 69 (44%) treated with carbapenem, and 87 (56%) with non carbapenem empiric therapy (36 patients treated with 3GC + aminoglycoside, 56 patients treated with PTZ + aminoglycoside) (Fig. 1).

Patients’ baseline characteristics are summarized in Table 1. Baseline characteristics were similar overall between patients receiving carbapenem and those receiving non carbapenem therapy, i.e., the rate of immunocompromised patients in both groups (respectively, 26 (38%) vs 32 (37%), *p* = 0.91) or the Charlson Index (respectively, 4 [2;6], and 3 [1.5;5], *p* = 0.37). Nevertheless, patients treated with non carbapenem therapy had more often cardiovascular history (70/87 (80%) vs 42/69 (61%), *p* = 0.01) but less history of urinary tract infections (41/87 (47%) vs 45/69 (65%), *p* = 0.02) particularly regarding ESBL-E infections (7/87 (17%) vs 28/69 (62%), *p* < 0.001) (Table 1).

**Table 1:** Patients’ characteristics, comorbidities, clinical, and biological presentation

	Total (*n* = 156)*	Carbapenem therapy (*n* = 69)	Non-carbapenem therapy (*n* = 87)	*p*-value
Characteristics at admission to ICU				
Age	69 [60;75]	68 [58;74]	70 [61;71]	0.17
Sex (male)	96 (61)	45 (65)	51 (59)	0.40
SAPSII	58 [52;67]	63 [55;71]	54 [46;65]	< 0.001
SOFA score	8 [7;10]	8 [7;10]	8 [6;10]	0.56
Comorbidities				
Diabetes mellitus	47 (30)	19 (27)	28 (32)	0.53
Chronic cardiac disease	112 (72)	42 (61)	70 (80)	0.01
Chronic respiratory disease	37 (24)	19 (27)	18 (21)	0.32
Chronic kidney disease	52 (33)	28 (41)	24 (28)	0.09
Chronic digestive disease	61 (39)	24 (35)	37 (42)	0.32
Chronic neurologic disease	76 (49)	32 (46)	44 (51)	0.60
Previous urinary tract infections UTI	86 (55)	45 (65)	41 (47)	0.02
History of ESBL-E UTI	35 (41)	28 (62)	7 (17)	< 0.001
Chronic urologic disease	111 (71)	50 (72)	61 (70)	0.75
Urologic neoplasia	32 (20)	13 (19)	19 (22)	0.59
Nephrolithiasis	27 (17)	10 (14)	17 (19)	0.36
Prior genitourinary surgery	49 (31)	22 (32)	27 (31)	0.96
Neurologic bladder	24 (15)	10 (14)	14 (16)	0.74
Urinary tract materials	55 (35)	24 (35)	31 (36)	0.91
Immunodepression	58 (37)	26 (38)	32 (37)	0.91
Risk factors for ESBL-E infection	143 (92)	67 (97)	76 (87)	0.03
Hospitalization in the past 3 months	127 (81)	60 (87)	67 (77)	0.11
Antibiotics in the past 3 months	96 (61)	55 (80)	41 (47)	< 0.001
Trip to an endemic area in the past 3 months	5 (3)	3 (4)	2 (2)	0.66
UTI or colonization with ESBL-E in the past 3 months	33 (21)	28 (40)	5 (6)	< 0.001
Nosocomial infection	126 (81)	58 (84)	68 (79)	0.35
Charlson comorbidity index	3 [2;5]	4 [2;6]	3 [1.5;5]	0.37
ICU length of stay (days)	5 [3;8]	5 [4;10]	4 [3;7.5]	0.98
Hospital length of stay (days)	15 [10;27.5]	15 [10;27]	15 [9;27]	0.71
Clinical and biological presentation				
Acute kidney injury	134 (86)	64 (93)	70 (80)	0.03
KDIGO stage I	25 (16)	10 (14)	15 (17)	
KDIGO stage II	44 (28)	17 (25)	27 (31)	
KDIGO stage III	65 (42)	37 (54)	28 (32)	
Septic cardiomyopathy^#^	36 (24)	19 (27)	17 (19)	0.24
Acute Respiratory Distress Syndrome	42 (27)	26 (38)	16 (18)	0.92
Stage I (PaO_2_/FiO_2_ = 200–300)	9 (21)	5 (19)	4 (25)	
Stage II (PaO_2_/FiO_2_ = 100–200)	17 (40)	11 (42)	6 (37)	
Stage III (PaO_2_/FiO_2_ < 100)	16 (38)	10 (38)	6 (37)	
Lactates (mmol/L)	3.8	3.8	3.7	0.70
WBC count (× 10^9^ cells/mm^3^)	18 [12–24]	17 [12–23]	19 [12–28]	0.36
Neurotrophic polynuclear cells (× 10^9^ cells/L), *n* = 143/156	15 [10–23]	14 [10–21]	16 [10–27]	0.21
Thrombocytopenia (< 150,000/mm^3^)	76 (49)	35 (51)	41 (47)	0.66
Platelets (× 10^9^ cells/mm^3^)	128 [74–224]	110 [60–220]	136 [84–230]	0.58
Hemoglobin (g/dL)	10.1 [8.7–11.5]	10 [8.6–11.5]	10.5 [8.8–11.3]	0.86
Natremia (mmol/L)	136 [132–140]	136 [132–140]	136 [131–140]	0.44
Kalemia (mmol/L)	4 [3.6–4.7]	4 [3.7–4.8]	4 [3.6–4.6]	0.69
CRP (mg/L), *n* = 78/156	187 [120–298]	214 [128–285]	180 [120–306]	0.42
PCT (ng/mL), *n* = 62/156	51 [15–98]	75 [15–124]	41 [15–80]	0.21

Results are presented as proportion (percentage) for categorical variables and median [interquartile range] for continuous variables

*p*-values are for comparison between carbapenem group and non carbapenem group. Threshold for statistical significance: *p* = 0.05

ARDS: Acute respiratory distress syndrome; CRP: C-reactive protein; ESBL-E: Extended-spectrum β-lactamase-producing *Enterobacterales*; ICU: intensive care unit; KDIGO: Kidney Disease Improving Global Outcomes; PCT: Procalcitonin; SAPS: Simplified Acute Physiology Score; SOFA: Sequential Organ Failure Assessment; UTI: urinary tract infection; WBC: white blood cell

^*^No missing data except when indicated

^#^Septic cardiomyopathy was defined by an elevated troponin + transthoracic echocardiography showing left ventricular ejection fraction < 40% + no significant coronary abnormalities in case of angiography

### Patients’ presentation

SAPS2 was significantly higher in the carbapenem group than in the non carbapenem group (respectively, 54 [46;65] vs 63 [55;71], *p* < 0.001) but not the SOFA score (8 [7;10] vs 8 [6;10], *p* < 0.56). Regarding organ failure, there was no significant difference in the rate of respiratory failure nor in the rate of cardiac impairment, but more patients in the carbapenem group had acute kidney injury (64/69 (93%) vs 70/87 (80.4%), *p* = 0.03) (Table 1). In the non carbapenem group, there were significantly more patients with lithiasis on urinary tract imaging (respectively, 11/69 (22%) vs 19/87 (16%), *p* = 0.05) (Additional file 1).

### Source control

Regarding the need for urological surgery, the two groups were comparable (29/69 (39%) vs 39/87 (45%), *p* = 0.47). All patients with urinary tract dilatation, regardless of the cause (lithiasis or non-lithiasis), underwent urinary diversion by catheterization (Additional file 2).

### Antimicrobial therapy

All patients in the non carbapenem group received aminoglycosides (78/87 received amikacin and 9/87 gentamycin). Only 5/69 patients of the carbapenem group did not receive aminoglycosides (61 received amikacin and 3 received gentamycin). 3GC therapy (*n* = 36) included cefotaxime (*n* = 25) and ceftriaxone (*n* = 11). Between H24 and H72, ESBL could be suspected either during the laboratory identification process or following information delivered by the family physician about ESBL carriage: 22 of the 56 patients receiving an empirical TZP treatment were switched to carbapenems, TZP was maintained in 18, and 6 received cotrimoxazole, 6 fluoroquinolones, and 4 cefoxitin. Another 31 patients received 3GC whom 9 were switched to carbapenems, 10 TZP, 5 fluoroquinolones, 4 cotrimoxazole, and 3 to cefoxitin. In the carbapenem group, 5 were switched to TZP, 3 cotrimoxazole, and 2 to fluoroquinolones. Total duration of antimicrobial treatment was 14 days [14;14] for carbapenem group and 14 days [14;15] for non carbapenem group (*p* = 0.5) (Table 2). Regarding aminoglycosides, 64/69 (93%) of patients receiving meropenem also received aminoglycosides infusion (57 (89%) 1 dose, 6 (9%) 2 doses, and 1 (2%) 3 doses); 56/56 (100%) of patients receiving TZP also received aminoglycosides (49 (88%) 1 dose, 5 (9%) 2 doses, and 2 (3%) 3 doses); and 31/31 (100%) of patients receiving 3CG also received aminoglycosides (25 (81%) 1 dose, 4 (13%) 2 doses, and 2 (6%) 3 doses). β-lactam therapy was never discontinued even in the case of susceptibility to aminoglycosides.

**Table 2:** Patients’ microbiological presentation, bacterial species, and antibiotic susceptibility test

	Total (*n* = 156)	Carbapenem therapy (*n* = 69)	Non-carbapenem therapy (*n* = 87)	*p*-value
Microbiological presentation				
Blood cultures performed	145 (93)	63 (91)	82 (94)	0.69
Concomitant bloodstream infection	109 (75)	52 (82)	57 (69)	0.07
Coinfection	6 (4)	3 (4)	3 (3)	1
ESBL fecal carriage	50 (32)	21 (30)	29 (33)	0.31
Bacterial species				
* Escherichia coli*	79 (51)	33 (48)	46 (53)	0.73
* Klebsiella pneumoniae*	46 (29)	22 (32)	24 (27)	
* Enterobacter cloacae*	15 (10)	8 (12)	7 (8)	
* Klebsiella oxytoca*,	4 (2)	2 (3)	2 (2)	
* Proteus mirabilis*	4 (2)	2 (3)	2 (2)	
* Citrobacter koseri*	3 (2)	2 (3)	1 (1)	
* Citrobacter freundii*	3 (2)	0 (0)	3 (3)	
* Morganella morganii*	2 (1)	0 (0)	2 (2)	
Antibiotic susceptibility test				
Susceptibility to piperacillin–tazobactam	90 (60)	33 (48)	57 (65)	0.06
Susceptibility to aminoglycoside	142 (91)	62 (90)	80 (92)	0.91
Susceptibility to piperacillin–tazobactam and/or aminoglycoside	148 (95)	65 (94)	83 (95)	1
Susceptibility to cefoxitin	91 (58)	37 (54)	54 (62)	0.06
Susceptibility to ertapenem, *n* (%)	148 (95)	64 (93)	84 (96)	0.45
Susceptibility to fluoroquinolone, *n* (%)	20 (13)	7 (10)	13 (15)	0.47
Susceptibility to cotrimoxazole/trimethoprim, *n* (%)	46 (29)	23 (33)	23 (26)	0.38

Results are presented as proportion (percentage) for categorical variables and median [interquartile range] for continuous variables

*p*-values are for comparison between carbapenem group and non carbapenem group. Threshold for statistical significance: *p* = 0.05

### Microbiological considerations

*Escherichia coli* and *K. pneumoniae* were the two most frequently isolated bacteria (52% and 30%, respectively). Most patients had associated bloodstream infections (52/69 (75%) in the carbapenem group vs 57/87 (65%) in the non carbapenem group, *p* = 0.07). Thirty-three out of 69 (48%) of the isolates were susceptible to PTZ (CMI ≤ 8 mg/L) in the carbapenem group vs 65/89 (65%) in the non carbapenem group (*p* = 0.06) and 62/69 (90%) and 80/87 (92%) were susceptible to aminoglycosides, respectively. Adequate empirical antimicrobial therapy was not different between non carbapenem (95%) and carbapenem groups (respectively, 95% vs 94%, *p* = 1.0) (Table 2). In the non carbapenem group, survivors and non-survivors had no difference regarding the proportion of susceptible isolated bacteria: (53/81 (65%) vs 4/6 (66%), *p* = 1.00) were TZP susceptible and (75/81 (93%) vs 5/6 (83%), *p* = 0.40) were susceptible to aminoglycosides. All the isolated bacteria were susceptible to the combination of TZP and aminoglycosides.

### Primary outcome and propensity score

After adjustment to the weighted propensity score (Additional file 3), there was no significant difference in mortality at Day 30 between the patients receiving carbapenem (10/69, (14%)) or non carbapenem therapy (6/87, (7%)) as empiric antimicrobial therapy (OR = 0.50 95% CI [0.19–1.80], *p* = 0.16).

### Secondary outcomes

**Fig. 2:**
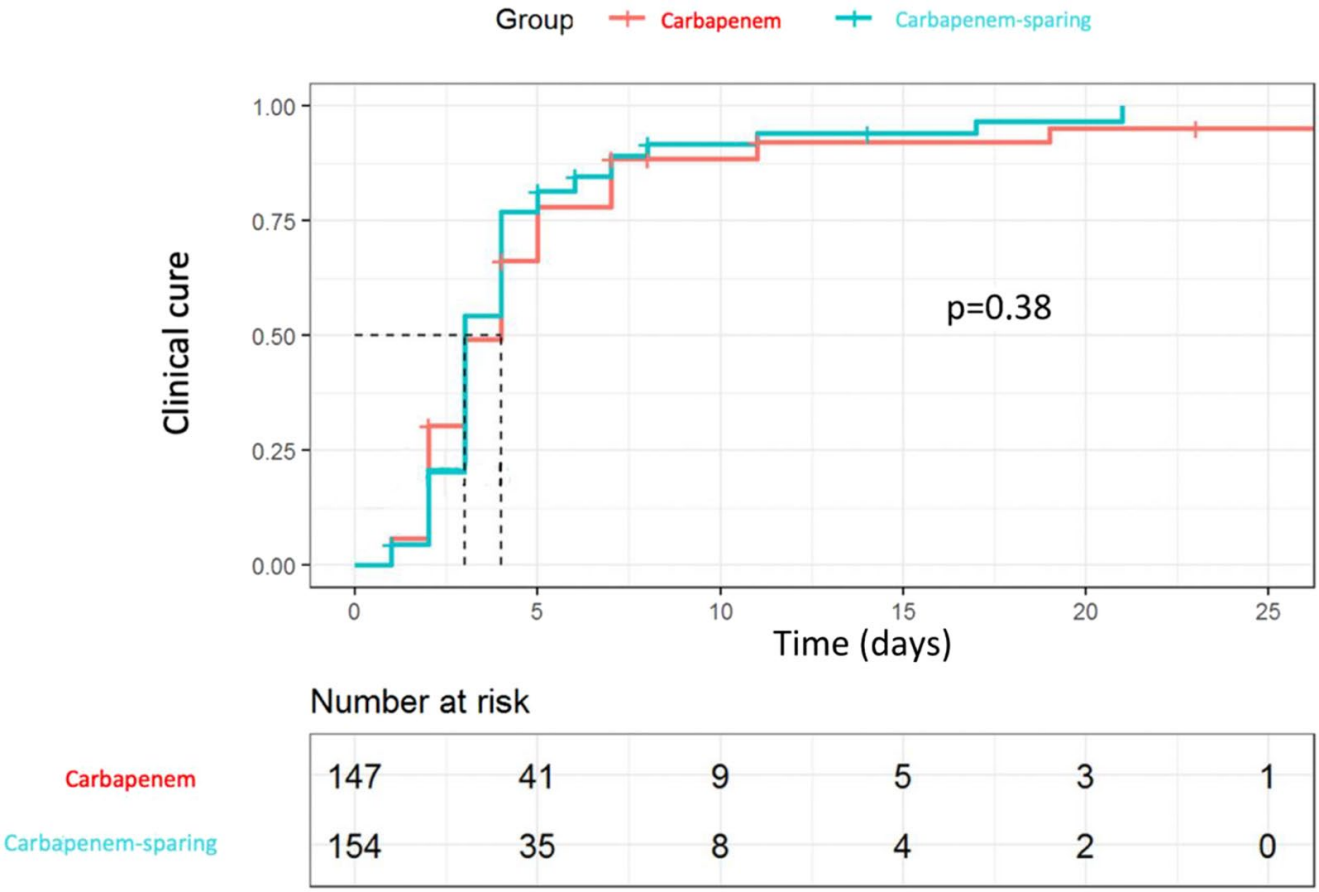
Kaplan–Meier curves of reflecting the probability of clinical cure according to the empirical antimicrobial therapy group (carbapenem vs non carbapenem regimen). Kaplan–Meier curves was weighted with the propensity score. For patients with treatment failure, data were censored for length of hospitalization. *P*-value results from the Log Rank Test

There was no significant difference regarding in-ICU mortality rate, Day-90 mortality, ICU length of stay, and hospital length of stay (Table 3). The use of renal replacement therapy was comparable in both groups, as was the need for invasive mechanical ventilation or vasopressive support with dobutamine. Maximal dose and duration of vasopressive support with norepinephrine were similar between the two groups. Time to clinical cure assessed by the Kaplan–Meier curve showed no difference between the two groups (3.8 days in the carbapenem group vs 3.3 days in the non carbapenem group, log rank test, *p* = 0.38) (Fig. 2). No difference in superinfection rates was observed between carbapenem and non carbapenem groups (*n* = 7; 10% vs *n* = 15; 17%; *p* = 0.56), respectively. No interaction in D30 mortality Odds ratios was observed between the subgroup of patients infected with PTZ R (OR 0.74; 95% CI 0.07–8.56) or S (OR 1.11; 95% CI 0.28–4.37) strains (*p* = 0.18). Only 1/36 patients treated with 3GC + aminoglycoside died.

**Table 3:** Outcomes

Outcome	Total (*n* = 156)	Carbapenem therapy (*n* = 69)	Non carbapenem therapy (*n* = 87)	OR	**β**	95% CI (OR or β)	*p*-value
Primary outcome							
D-30 mortality, *n* (%)	16 (10)	10 (15)	6 (7)	0.5	0.19;1.80		0.14
Secondary outcomes							
ICU mortality, *n* (%)	20 (13)	11 (16)	9 (10)	0.7		0.26;1.88	0.42
Day-90 mortality, *n* (%)	25 (16)	12 (17)	13 (15)	0.9		0.38;2.30	0.90
ICU length of stay (days), median ± IQR	5 [3;8]	5 [4;10]	4 [3;7.5]		−0.26	−4.52;4.44	0.90
Hospital length of stay (days), median ± IQR	15 [10;27.5]	15 [10;27]	15 [9;27]		−0.85	−7.75;6.85	0.82
Mechanical ventilation, *n* (%)	45 (30)	24 (35)	21 (24)	0.5		0.28;1.08	0.09
Renal replacement therapy, *n* (%)	25 (16)	14 (20)	11 (13)	0.5		0.22;1.21	0.10
Total duration norepinephrine (hours), median ± IQR	48 [36–72]	48 [36–72]	48 [36–72]		5.4	−11.34;22.21	0.52
Maximal dose norepinephrine (gamma/kg/min), median ± IQR	0.4 [0.2–0.7]	0.5 [0.3–0.7]	0.4 [0.3–0.6]		−0.17	−0.39;0.03	0.09

For all secondary judgment criteria, the parameter estimates of the regression models were estimated by bootstrapping. The models were weighted based on the final propensity score. *p*-values are for comparison between carbapenem group and non carbapenem group. Threshold for statistical significance: *p* = 0.05

Results are presented as proportion (percentage) for categorical variables and median [interquartile range] for continuous variables

## Discussion

In this original study of ESBL-E urinary septic shock patients, no difference in Day-30 mortality and time to clinical cure was observed in patients treated with a non carbapenem empiric therapy compared with those treated with carbapenems. This is the first study showing the potential efficacy of non carbapenem therapy as empiric treatment in ESBL-E urinary tract septic shock. Treatment of infections with ESBL-E remains a challenge extensively discussed in the literature, but very few data regarding urinary septic shock patients were reported to date.

The current French guidelines [21] recommend the use of carbapenems whenever there is a risk factor for infection with an ESBL-E. Nevertheless, those risk factors were determined in studies conducted in countries with high ESBL-E prevalence and mostly in retrospective studies [22]. The INCREMENT cohort compared empiric treatment with non carbapenem agents (86 patients mainly aminoglycosides (*n* = 42) and fluoroquinolones) vs carbapenems (245 patients). No significant difference in 30-day mortality between those two groups was observed [23]. Another retrospective study compared empiric therapy with aminoglycosides *vs* carbapenems or PTZ for the treatment of ESBL-E UTIs, demonstrating the non-inferiority of aminoglycosides in terms of 30-day mortality. However, these studies involved only a low rate of septic shock [17]. In our study, the role of systematically administrated aminoglycosides along with carbapenem or non carbapenem therapy is probably important as they are known to have a good penetration of urinary tract and as shown by the low mortality rate of patients treated with 3 GC + aminoglycoside.

PTZ is another interesting candidate as a carbapenem-sparing agent. Yoon et al. compared the clinical efficacy of PTZ vs ertapenem and showed no significant difference regarding microbiological eradication failure (respectively, 4.4% vs 4.9%, *p* = 1.00) and in-hospital mortality (respectively, 4.4% vs 13.4%, *p* = 0.06) [24]. Another multicenter retrospective propensity-matched control study included 186 patients with ESBL-E urinary tract infections, 27% of whom admitted in ICU and found no difference in the resolution of clinical symptoms by Day 7 or in 30-day mortality [25]. Several retrospective cohort studies compared the efficacy of PTZ to carbapenems as empiric antimicrobial therapy in ESBL-E bloodstream infections with neither difference regarding mortality nor clinical response [23]. Another French retrospective multicenter study, including about 100 severe ESBL infections, did not find any significant difference between a combination of beta-lactamase inhibitors and carbapenems on mortality at D30, but only 6% of these infections were urinary tract infections [11]. Finally, in a meta-analysis including 35 publications, no significant differences in overall mortality between a carbapenem *vs* a non carbapenem regimen was showed [26].

The only randomized trial investigating the non-inferiority of PTZ vs meropenem on Day-30 mortality in the treatment of 3GC-resistant *E. coli* and *K. pneumoniae* bloodstream infection (UTI in 60% of the patients) did not prove non-inferiority of a carbapenem-sparing strategy, including PTZ, compared with meropenem (Day-30 mortality, respectively, 12.3% and 3.7% (CI 97.5% [− ∞; + 14.5] *p* = 0.9)) [12], but some critics have cautioned the authors’ conclusions that PTZ was inferior and carbapenems therefore recommended [10].

In our study, Day-30 mortality is estimated at 10%, which is clearly below the rates reported by the various descriptive epidemiological studies in France and Europe [13]. This could be explained by the fact that mortality is lower in patients with septic shock from urinary tract compared to other sources. The optimization of the management of septic shock since the Sepsis Survey Campaign can also have played a role. Finally, it is suggested that virulence and resistance could possibly be antagonistic in Gram-negative bacteria especially *Escherichia coli* [27].

The major limitation of this study is its retrospective design limiting the ability to draw definitive conclusions. Nevertheless, we used a weighted propensity-matched analysis to decrease the impact of potential confounding factors. Our study focuses only on ESBL-E, which differs from the Merino cohort, which also included C3G-resistant strains through production of AmpC cephalosporinase, which has a direct impact on PTZ susceptibility. In addition, MICs for TZP were within the EUCAST guidelines of 8 mg/L or less, and strains classified as intermediate were considered resistant in this study. Regarding the percentages of *Escherichia coli* and *Klebsiella pneumoniae* isolates susceptible to PTZ and aminoglycosides, our results were in agreement with epidemiological studies [7]. Moreover, in both groups, mortality at Day30 was not related to urinary septic shock treatment failure but rather to complications of ICU stay. Besides, the absence of difference in D30 mortality in the non carbapenem group could be explained by the substantial number of patients in the non carbapenem group who were switched to carbapenem as soon as ESBL was suspected. Finally, this study had not sufficient power to detect small differences, such as the rate of superinfections, which was slightly higher in the non carbapenem group (17 vs 10%) without statistical significant difference.

## Conclusions

In ESBL-E urinary septic shock, when the empirical treatment include an aminoglycoside and is thus adequate, the 30-day mortality of patients was not different whatever the β-lactam used (carbapenem or non carbapenem).Therefore, our results strongly encourage for future large multicenter prospective non-inferiority randomized study aiming at confirming that a strategy, including non carbapenem β-lactam and aminoglycoside, is safe alternatives to carbapenems as empirical antimicrobial therapy in ESBL-E urinary septic shock.

## Supplementary Information

**Additional file 1.** Patients’ imaging presentation.

**Additional file 2.** Interventions, source control and antimicrobial therapy.

**Additional file 3.** Distribution of the propensity to receive carbapenem before vs after adjustment to the propensity score. Patients’ microbiological presentation, bacterial species and antibiotic susceptibility test.

**Additional file 4.** Standardized differences before/after adjustment by weighting (95% CI).

## Data Availability

The datasets analyzed during the current study are available from the corresponding author on reasonable request.

## Abbreviations

2GC: 2nd-generation cephalosporin
3GC: 3rd-generation cephalosporin
AKI: Acute kidney injury
ARDS: Acute respiratory distress syndrome
βL-βLIs: β-lactam–β-lactamase inhibitors
CNIL: Commission Nationale de l'Informatique et des Libertés
CRE: Carbapenem-resistant *Enterobacterales*
ESBL: Extended-spectrum β-lactamase
ESBL-E: Extended-spectrum β-lactamase—*Enterobacterales*
EUCAST: European Committee on Antimicrobial Susceptibility Testing
HAS: Haute Autorité de Santé
ICU: Intensive care unit
IQR: Inter quartile range
KDIGO: Kidney Disease Improving Global Outcomes
MDR: Multi-drug Résistant
MIC: Minimum Inhibitory Concentration
ONERBA: Observatoire National de l’Epidémiologie de la Résistance aux Antibiotiques
PMSI: Programme de Médicalisation des Systèmes d’Information
PTZ: Piperacillin/Tazobactam
SAPS: Simplified acute physiology score
SOFA: Sequential Organ Failure Assessment
SRLF: Société de Réanimation de Langue Française
UTI: Urinary tract infection
WHO: World Health Organization
