# Supplementary material for: Efficacy of carbapenem vs non carbapenem β-lactam therapy as empiric antimicrobial therapy in patients with extended-spectrum β-lactamase-producing Enterobacterales urinary septic shock: a propensity-weighted multicenter cohort study
Source: Ann Intensive Care. 2023 Mar 24;13:22. doi: 10.1186/s13613-023-01106-z (PMC10036246; doi:10.1186/s13613-023-01106-z)
Supplement: Supplementary file 1 — Additional file 1. Patients’ imaging presentation. [file 13613_2023_1106_MOESM1_ESM.docx]

Additional file 1. Patients’ imaging presentation

|  | Total (n=156) | Carbapenem therapy  (n= 69) | Carbapenem-sparing therapy (n=87) | p-value | |
| --- | --- | --- | --- | --- | --- |
| **Imaging** | | | | |  |
| Pathologic imaging test n (%) | 91 (58) | 43 (62) | 48 (55) | 0.37 |  |
| Urinary tract dilatation n (%) | 67 (43) | 31 (45) | 36 (42) |  |  |
| Lithiasis n (%) | 30 (19) | 11 (16) | 19 (21) |  |  |
| Peri-renal infiltration n (%) | 48 (31) | 26 (38) | 22 (25) |  |  |
| Abscess n (%) | 15 (10) | 9 (13) | 6 (7) |  |  |

P-values are for comparison between carbapenem group and carbapenem-sparing group.
