# Supplementary material for: Efficacy of carbapenem vs non carbapenem β-lactam therapy as empiric antimicrobial therapy in patients with extended-spectrum β-lactamase-producing Enterobacterales urinary septic shock: a propensity-weighted multicenter cohort study
Source: Ann Intensive Care. 2023 Mar 24;13:22. doi: 10.1186/s13613-023-01106-z (PMC10036246; doi:10.1186/s13613-023-01106-z)
Supplement: Supplementary file 2 — Additional file 2. Interventions, source control and antimicrobial therapy. [file 13613_2023_1106_MOESM2_ESM.docx]

Additional file 2. Interventions, source control and antimicrobial therapy.

|  | Total (n=156) | Carbapenem therapy  (n= 69) | Non carbapenem therapy (n=87) | p-value |
| --- | --- | --- | --- | --- |
| **Interventions** | | | | |
| Mechanical ventilation, n (%) | 45 (29) | 24 (35) | 21 (24) | 0.14 |
| Renal replacement therapy, n (%) | 25 (16) | 14 (20) | 11 (13) | 0.20 |
| Duration of norepinephrine (hours), mean | 65 | 63 | 66 | 0.81 |
| Maximal dose of norepinephrine (µg/kg/min), mean | 0.6 | 0.7 | 0.5 | 0.06 |
| Use of dobutamine, n (%) | 20 (13) | 12 (17) | 8 (9) | 0.13 |
| **Source control** | | | | |
| Urologic surgery, n (%) | 68 (44) | 29 (42) | 39 (45) | 0.47 |
| Urinary diversion, n (%) | 67 (43) | 28 (40.5) | 39 (45) |  |
| Abscess surgery, n (%) | 2 (1.3) | 1 (1.4) | 1 (1.1) |  |
| **Antimicrobial therapy** | | | | |
| Antibiotic therapy in accordance with the current recommendations, n (%) | 78 (50) | 67 (97) | 11 (13) | <0.001 |
| Potential sparing of carbapenems according to susceptibility testing, n (%) | 148 (95) | 65 (94) | 83 (95) | 1 |
| Total duration of antimicrobial therapy (days), median (IQR) | 14 [14;15] | 14[14;14] | 14[14;15] | 0.50 |
| *Clostridiale difficile* infection, n (%) | 2 (1) | 1 (1) | 1 (1) | 1 |
| Recurrence of an ESBL-E infection, n (%) | 24 (15) | 7 (10) | 17 (20) | 0.56 |
| Carbapenemase-producing bacterial infection, n (%) | 1 (0.6) | 1 (1.5) | 0 (0) | 0.44 |
