# Supplementary material for: Efficacy of carbapenem vs non carbapenem β-lactam therapy as empiric antimicrobial therapy in patients with extended-spectrum β-lactamase-producing Enterobacterales urinary septic shock: a propensity-weighted multicenter cohort study
Source: Ann Intensive Care. 2023 Mar 24;13:22. doi: 10.1186/s13613-023-01106-z (PMC10036246; doi:10.1186/s13613-023-01106-z)
Supplement: Supplementary file 3 — Additional file 3. Distribution of the propensity to receive carbapenem before vs after adjustment to the propensity score. Patients’ microbiological presentation, bacterial species and antibiotic susceptibility test. [file 13613_2023_1106_MOESM3_ESM.docx]

Additional file 3. Distribution of the propensity to receive carbapenem before *vs* after adjustment to the propensity score.


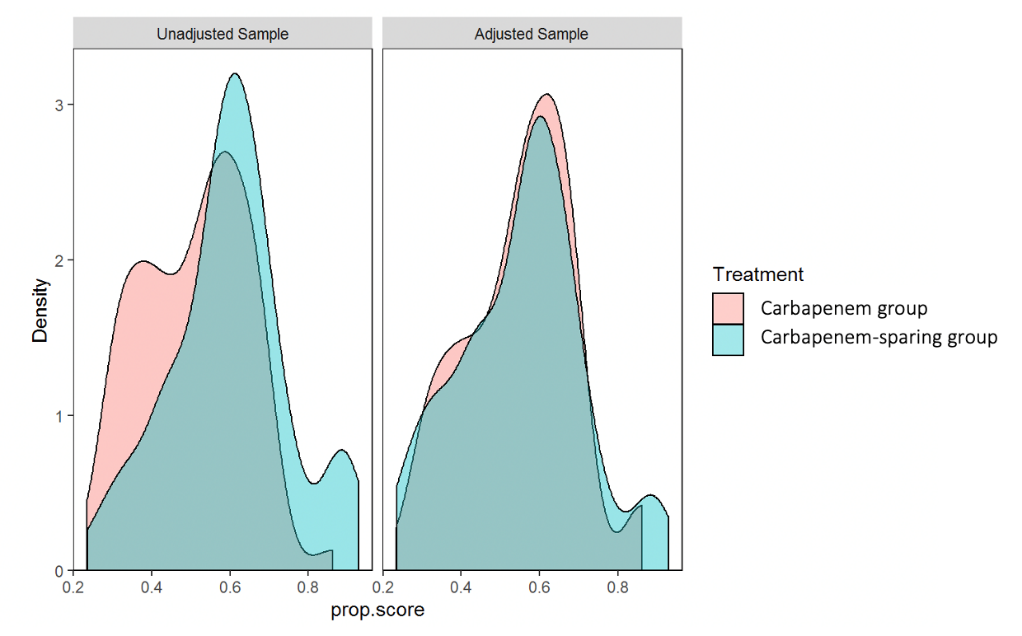


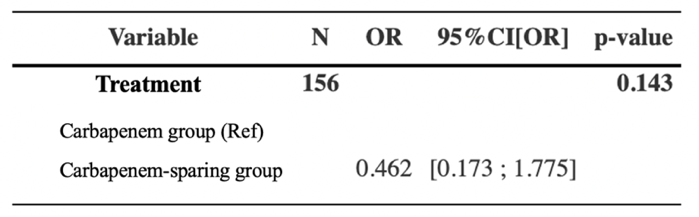


Additional file 3. Patients’ microbiological presentation, bacterial species and antibiotic susceptibility test.

|  | Total (n=156) | Carbapenem therapy  (n= 69) | Carbapenem-sparing therapy (n=87) | p-value |
| --- | --- | --- | --- | --- |
| **Microbiological presentation** | | | | |
| Blood cultures performed, n (%) | 145 (93) | 63 (91) | 82 (94) | 0.69 |
| Concomitant bloodstream infection, n (%) | 109 (75) | 52 (82) | 57 (69) | 0.07 |
| Coinfection, n (%) | 6 (4) | 3 (4) | 3 (3) | 1 |
| ESBL faecal carriage, n (%) | 50 (32) | 21 (30) | 29 (33) | 0.31 |
| **Bacterial species** | | | | |
| *Escherichia coli*, n (%) | 79 (51) | 33 (48) | 46 (53) | 0,73 |
| *Klebsiella pneumoniae*, n (%) | 46 (29) | 22 (32) | 24 (27) |  |
| *Enterobacter cloacae*, n (%) | 15 (10) | 8 (12) | 7 (8) |  |
| *Klebsiella oxytoca*, n (%) | 4 (2) | 2 (3) | 2 (2) |  |
| *Proteus mirabilis*, n (%) | 4 (2) | 2 (3) | 2 (2) |  |
| *Citrobacter koseri*, n (%) | 3 (2) | 2 (3) | 1 (1) |  |
| *Citrobacter freundii*, n (%) | 3 (2) | 0 (0) | 3 (3) |  |
| *Morganella morganii*, n (%) | 2 (1) | 0 (0) | 2 (2) |  |
| **Antibiotic susceptibility test** | | | | |
| Susceptibility to piperacillin-tazobactam, n (%) | 90 (60) | 33 (48) | 57 (65) | 0.06 |
| Susceptibility to aminoglycoside, n (%) | 142 (91) | 62 (90) | 80 (92) | 0.91 |
| Susceptibility to piperacillin-tazobactam and/or aminoglycoside, n (%) | 148 (95) | 65 (94) | 83 (95) | 1 |
| Susceptibility to cefoxitin, n (%) | 91 (58) | 37 (54) | 54 (62) | 0.06 |
| Susceptibility to ertapenem, n (%) | 148 (95) | 64 (93) | 84 (96) | 0.45 |
| Susceptibility to fluoroquinolone, n (%) | 20 (13 | 7 (10) | 13 (15) | 0.47 |
| Susceptibility to cotrimoxazole / trimethoprim, n (%) | 46 (29) | 23 (33) | 23 (26) | 0.38 |
