# Supplementary figures and images for: Efficacy of carbapenem vs non carbapenem β-lactam therapy as empiric antimicrobial therapy in patients with extended-spectrum β-lactamase-producing Enterobacterales urinary septic shock: a propensity-weighted multicenter cohort study
Source: Ann Intensive Care. 2023 Mar 24;13:22. doi: 10.1186/s13613-023-01106-z (PMC10036246; doi:10.1186/s13613-023-01106-z)

Additional file 4.

Standardized differences before/after adjustment by weighting (95% CI).


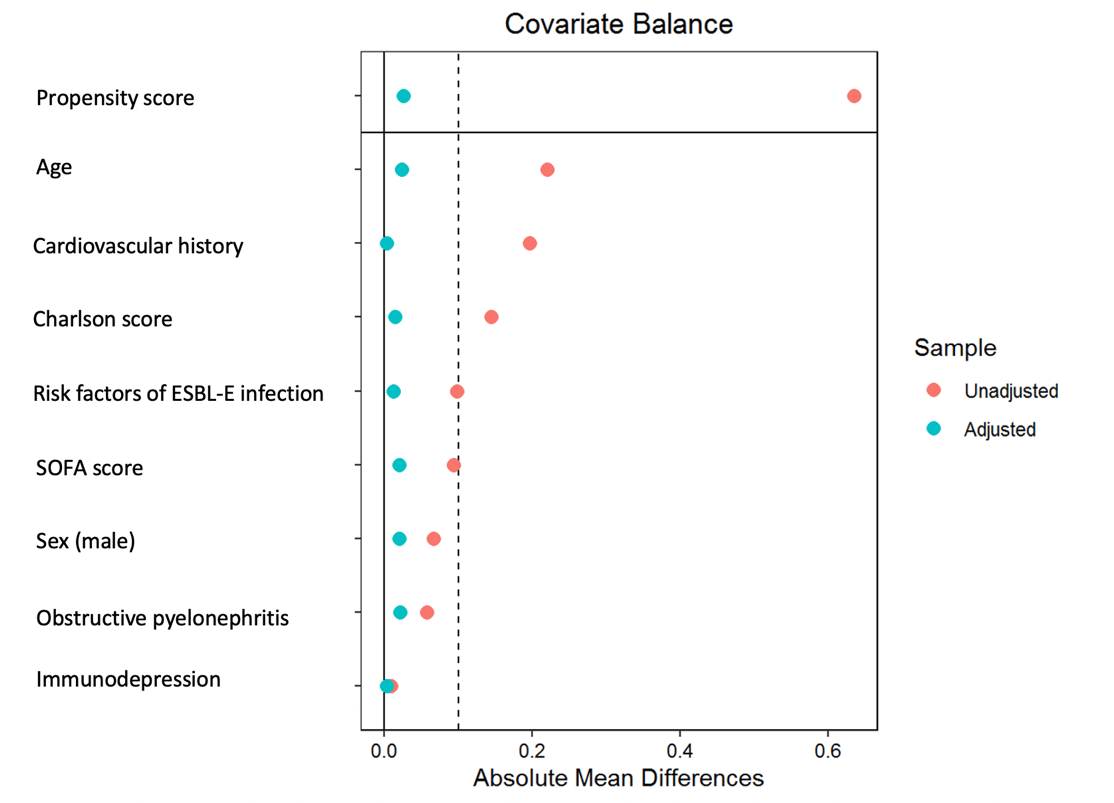

Supplement: Supplementary file 4 — Additional file 4. Standardized differences before/after adjustment by weighting (95% CI). [file 13613_2023_1106_MOESM4_ESM.docx]
